# Surgeon John B. Fontaine

**Published:** 1864-10

**Authors:** 


### OBITUARY.

*Surgeon John B. Fontaine.*

KILLED, in the line of duty, rendering professional service to General Dunnovant, on the battle-field near Petersburg, October 1st, 1864, Surgeon JOHN B. FONTAINE, Medical Director, Cavalry Corps, Army Northern Virginia, aged 24 years.

Few words serve to record this event—but no language can describe the grievous shock to parents and family: to widowed wife and mourning friends who weep around the early grave of our professional brother.

Short, but brilliant has been his career. Graduating at the Medical College of Virginia in 1860, he was among the first to enter the army, and remained in the field, on active duty, until death found him—the chief medical officer of the corps to which he had always been attached—respected, beloved by all.

Knowing his ardent and gallant spirit, it was feared that his valuable life had been lost unnecessarily, for often was he foremost on the scout, eager in the charge, hot in pursuit; but kind Providence spared him for that better moment when in ministering to the wants of a dying soldier, he received a mortal wound.

It has been well said, that man's life should be measured, not by its length, but its breadth. Why then do we mourn the death of our friend? Four years was long enough to prove him the accomplished physician, gallant soldier, true and honorable gentleman. Faithful as a son and brother, devoted husband and fond father, he has worthily run his course, and his spirit wings its upward flight to join the band of martyred heroes who have made the name and fame of their native land immortal.—ED.

